# Development of an *In Vivo* Probe to Track SARS-CoV-2 Infection in Rhesus Macaques

**DOI:** 10.3389/fimmu.2021.810047

**Published:** 2021-12-24

**Authors:** Patrick J. Madden, Muhammad S. Arif, Mark E. Becker, Michael D. McRaven, Ann M. Carias, Ramon Lorenzo-Redondo, Sixia Xiao, Cecily C. Midkiff, Robert V. Blair, Elizabeth Lake Potter, Laura Martin-Sancho, Alan Dodson, Elena Martinelli, John-Paul M. Todd, Francois J. Villinger, Sumit K. Chanda, Pyone Pyone Aye, Chad J. Roy, Mario Roederer, Mark G. Lewis, Ronald S. Veazey, Thomas J. Hope

**Affiliations:** ^1^ Department of Cell and Developmental Biology, Feinberg School of Medicine, Northwestern University, Chicago, IL, United States; ^2^ Department of Medicine, Division of Infectious Diseases, Northwestern University Feinberg School of Medicine, Chicago, IL, United States; ^3^ Center for Pathogen Genomics and Microbial Evolution, Northwestern University Institute for Global Health, Chicago, IL, United States; ^4^ Division of Comparative Pathology, Tulane National Primate Research Center, Covington, LA, United States; ^5^ Vaccine Research Center, National Institute of Allergy and Infectious Diseases, National Institutes of Health, Bethesda, MD, United States; ^6^ Immunity and Pathogenesis Program, Sanford Burnham Prebys Medical Discovery Institute, La Jolla, CA, United States; ^7^ Bioqual Inc., Rockville, MD, United States; ^8^ New Iberia Research Center, University of Louisiana-Lafayette, New Iberia, LA, United States; ^9^ Division of Microbiology, Tulane National Primate Research Center, Covington, LA, United States

**Keywords:** SARS-CoV-2, nonhuman primates, rhesus macaque, antibodies, COVID-19, antibody probes

## Abstract

Infection with the novel coronavirus, SARS-CoV-2, results in pneumonia and other respiratory symptoms as well as pathologies at diverse anatomical sites. An outstanding question is whether these diverse pathologies are due to replication of the virus in these anatomical compartments and how and when the virus reaches those sites. To answer these outstanding questions and study the spatiotemporal dynamics of SARS-CoV-2 infection a method for tracking viral spread *in vivo* is needed. We developed a novel, fluorescently labeled, antibody-based *in vivo* probe system using the anti-spike monoclonal antibody CR3022 and demonstrated that it could successfully identify sites of SARS-CoV-2 infection in a rhesus macaque model of COVID-19. Our results showed that the fluorescent signal from our antibody-based probe could differentiate whole lungs of macaques infected for 9 days from those infected for 2 or 3 days. Additionally, the probe signal corroborated the frequency and density of infected cells in individual tissue blocks from infected macaques. These results provide proof of concept for the use of *in vivo* antibody-based probes to study SARS-CoV-2 infection dynamics in rhesus macaques.

## Introduction

Since its emergence in late 2019, Severe Acute Respiratory Syndrome Coronavirus 2 (SARS-CoV-2) has spread throughout the world causing a global pandemic of Coronavirus Disease 2019 (COVID-19). COVID-19 is considered primarily a respiratory disease as the initial characterizations focused on the resulting pneumonia and respiratory symptoms of cough, difficulty breathing, low O_2_ saturation, and other lung pathologies (1, 2). However, it has become increasingly clear that people who have COVID-19 experience the effects of infection at multiple anatomical sites. Some well documented non-respiratory symptoms include brain fog, loss of taste and smell, kidney failure, and gastrointestinal symptoms (3–8). Additionally, there are reports of several other potential non-respiratory symptoms including erectile dysfunction and cardiovascular pathologies (9–11). The underlying question for all these symptoms is whether they are caused directly by infection at these sites or are secondary effects of infection elsewhere. Limited studies have shown the presence of virus in tissues outside of the respiratory tract, but these studies have used autopsy tissue or biopsies from infected individuals (3, 4, 7, 12). Unfortunately, these single time-point samples cannot be leveraged to understand the kinetics of how the infection spreads to these tissues. There is also some indication that SARS-CoV-2 can persist at specific anatomical sites, which could be related to long COVID symptoms (3, 13, 14). A better understanding of the spatiotemporal dynamics of SARS-CoV-2 infection would help to determine sites of viral persistence and elucidate the biology behind non-respiratory tract symptoms. However, both a model system that closely recapitulates human disease and a way to track infection *in vivo* is needed to study these spatiotemporal dynamics.

The rhesus macaque (*Macaca mulatta*) model of infection has been used extensively during the COVID-19 pandemic to study vaccines and therapeutics and to uncover the underlying biology of SARS-CoV-2 (15–24). After concomitant intra-nasal and intra-tracheal administration of SARS-CoV-2, rhesus macaques recapitulate viral shedding and pathologic signs of viral pneumonia in the lungs (15, 18–21, 23). Despite this, severe clinical outcomes seen in humans, such as acute respiratory distress syndrome, are rarely reproduced in most non-human primates (16, 23). However, non-human primates remain the ideal model to study SARS-CoV-2 pathogenesis due to their anatomical and immunological similarities to humans (23). In addition, animal models can be leveraged to study the kinetics of infections and to better analyze tissues during active viral replication when paired with *in vivo* probes.

Antibody-based *in vivo* probes can facilitate tracking of viral spread in an unbiased manner and uncover new anatomical sites of viral replication. These probes rely on an immunoglobulin G (IgG) targeting a specific protein of interest that is chemically modified to allow tracking and detection of the probe. Antibody-based probes can be labeled with radioisotopes detected using positron emission tomography (PET) or with fluorescent dyes detected through optical imaging. These techniques have been used for many years to image cancerous cells and biological processes, but only more recently have they been applied to study pathogens (25–34). Studies of fungi, bacteria, and even parasites have been undertaken using pathogen specific antibodies as probes to study the distribution and kinetics of infection (35–39). In addition, we and others have used imaging of antibody probes to study the dynamics of viral infection (40–42). However, unlike the pathogens mentioned above, viruses hijack the machinery of the cells they infect to replicate; this leads to viral proteins being expressed by infected cells. We can exploit this mechanism by using antibodies against viral proteins to uncover the location of infected cells *in vivo*. The utility of this approach, to describe novel sites of infection in an unbiased manner, has been demonstrated in simian immunodeficiency virus (SIV) infection (40, 42). Here, using a similar approach, we describe the development of the first SARS-CoV-2 specific antibody-based probe, the fluorescently tagged anti-spike IgG, CR3022, to study the spatiotemporal dynamics of SARS-CoV-2 infection. We find that following administration of this probe to SARS-CoV-2 infected rhesus macaques the probe penetrates the lung tissue and the corresponding fluorescent signal can be used to discern between areas of tissue with many infected cells versus areas with few or none. In addition, the probe’s ability to detect infection was greater after one week of infection compared to 2- or 3-days post infection (p.i.). These results provide proof of concept for using antibody-based probes to track viral spread and study the spatiotemporal dynamics of SARS-CoV-2 in the rhesus macaque model. Future use of radiolabeled versions of this probe targeting SARS-CoV-2 will overcome the limitations of fluorescent labeling. The unbiased, whole-body, PET/CT scan will provide unique insights into the spatiotemporal dynamics of SARS-CoV-2 distribution and dissemination providing novel insights into COVID-19 related pathology.

## Materials And Methods

### F(ab’)2 Production and Labeling

The monoclonal antibody CR3022 was purchased from Absolute Antibody (#Ab01680-10.0) as an IgG1. F(ab’)2 was produced using the Genovis FragIT kit (#A2-FR2-100, Genovis) according to manufacturer’s protocol. Briefly, 1-5 mg of IgG1 was added to the FragIT column after equilibration and allowed to rock at room temperature for 45 minutes, the column was then centrifuged to elute IgG fragments. Fc fragments were removed by passing through a CaptureSelect Fc Column. F(ab’)2 fragments were eluted and collected. F(ab’)2 production was checked through SDS-PAGE. The resulting gel was first imaged under white light to show the fluorophore labeling of the F(ab’)2 before staining with SimplyBlue SafeStain (Thermo-Fisher, #LC6060). For labeling, 3 mgs of purified CR3022-F(ab’)2 was combined with 60ug of sulfo-Cy5 NHS ester (#53320, Lumiprobe), sulfo-Cy3 NHS ester (#51320, Lumiprobe), or AF647-NHS ester (#A20106, Thermo-Fisher) in PBS with 100mM sodium bicarbonate and gently rocked at room temperature for 1 hour as previously described (43). Solutions were then passed through a Zeba column (Thermo-Fisher, #89882) to remove free dye. Labeled F(ab’)2 was filtered through a 0.22-μm filter and stored at 4°C in the dark. Labeled F(ab’)2 to be infused into macaques was tested using the Pierce LAL Chromogenic Endotoxin Quantitation Kit (Thermo-Fisher, #88282).

### *In Vitro* Probe Testing in 293T Cells

293T cells were transfected with a plasmid expressing the spike protein of the WA1 strain of SARS-CoV-2 (a gift from Tom Gallagher at Loyola University) using polyethyleneimine (Fisher Scientific, #AC1785710000). Twenty-four hours after transfection fresh media was added to the cells that contained the fluorescently labeled CR3022-F(ab’)2. Twenty-four hours after addition of the CR3022-F(ab’)2 the cells were fixed and stained with a rabbit anti-SARS-CoV-2 spike antibody (Sino Biological, #40150-R007) and Hoechst (1:25,000, Thermo-Fisher). The cells were imaged using a DeltaVision inverted light microscope and images were analyzed using softWoRx software (Applied Precision).

### *In Vitro* Probe Testing in Human Airway Epithelial Cultures

Human airway epithelium (HAE) cultures were produced according to previously established methods (44, 45). Briefly, primary bronchial epithelial cells from a single donor (#CC2540S, Lonza, Switzerland) were differentiated at an air-liquid interface in Pneumacult ALI medium (#05001, Stemcell Technologies, Canada) for at least 4 weeks. Differentiation was assessed by observation of ciliary motion and elevated transepithelial electrical resistance; cultures were used for experiments within 2 months of differentiation. HAE cultures were infected with SARS-CoV-2 WA1 at a multiplicity of infection (MOI = 1), as described previously, leaving the apical surface exposed to air after removal of the viral inoculum (45). Forty-eight hours after infection, CR3022-F(ab’)2-Cy3 was applied at 0.01 mg/mL (approximating macaque serum concentration) to the basolateral surface in media. At 72 hours post infection, cultures were fixed in 5% formaldehyde for 4 hours, then frozen in optimal cutting temperature (OCT) medium and sectioned for analysis by microscopy. Tissue sections were stained with a mouse-human chimeric anti-SARS-CoV-2 spike antibody (#40150-D003, Sino Biological, China) and Hoechst (1:25,000, Thermo-Fisher, USA). Experiments with SARS-CoV-2 were performed in a Biosafety Level 3 (BSL3) laboratory under the approval of the Sanford Burnham Prebys Medical Discovery Institute Biosafety Committee.

### Animals

A total of five rhesus macaques (*Macaca mulatta*) were used for probe development. Two macaques were housed at Bioqual, Inc. (Rockville, MD) and the remaining three were housed at the Tulane National Primate Research Center (TNPRC, Covington, LA). Both facilities are accredited by the Association for the Assessment and Accreditation of Laboratory Animal Care. All procedures performed at TNPRC were reviewed and approved by the Tulane University Institutional Animal Care and Use Committee (IACUC) under protocol numbers P0452 and P0447. All procedures performed at Bioqual, Inc. were reviewed and approved by the Bioqual, Inc. IACUC under protocol number 20-V865-052P.

Four animals were inoculated with 1.1x10^6^ tissue culture infectious dose (TCID_50_) of the WA1 strain of SARS-CoV-2 (2019-nCoV/USA-WA1/2020, BEI#NR-52281) *via* intratracheal/intranasal instillation (1mL intratracheal, 500µL per each nare), as previously described (17, 18). A single animal was left uninfected to serve as a naïve control. 24 hours prior to necropsy all five animals were infused intravenously (i.v.) with 0.5 mg/kg of CR3022-F(ab’)2 probe fluorescently labeled with Cy3, Cy5, or AF647. The uninfected control animal was infused with 0.5 mg/kg of each Cy3 and Cy5 labeled probe for a total of 1 mg/kg. Animals housed at TNPRC had nasal and pharyngeal swabs taken on days 1, 2, 3, 5 and at necropsy and had bronchoalveolar lavages (BAL) performed on days 1, 3, and at necropsy. All infected animals were necropsied 2- (LP86), 3- (DGD8) or 9-days (KF89 and LM30) p.i. At necropsy, all tissues were placed into 10% neutral buffered formalin for at least 72 hours before being removed from the biosafety level 3 laboratory. Fixed tissue samples were then shipped to Northwestern University for imaging.

### Quantification of Viral RNA

Viral load was quantified in pharyngeal and nasal swabs as well as supernatant and cells from BAL for the animals housed at TNPRC using RT-qPCR. Probes targeting the nucleocapsid or envelope gene were used to quantify both genomic and subgenomic RNA of SARS-CoV-2. A Zymo Quick RNA Viral Kit (#R1035 or #D7003), Zymo, USA) was used to isolate RNA according to manufacturer’s protocol and as previously described (17). RNA was analyzed using a QuantStudio 6 (Thermo Scientific, USA) and TaqPath master mix (Thermo Scientific, USA) as previously described (17).

### IVIS Imaging

Upon arrival at Northwestern University, tissues were washed with PBS then imaged on an IVIS Lumina Series III (Perkin Elmer) using the fluorescent imaging settings. Whole organs or large pieces of tissue were imaged using the same exposure conditions for each fluorophore, respectively (Cy3 and Cy5). The lungs were further separated into individual lobes and imaged again under identical conditions. Small pieces of tissue (~2x2cm^2^) were cut from whole organs and re-imaged on the IVIS before being frozen in OCT in cryomolds. Imaging conditions were kept consistent between animals that received the same fluorophore and all resulting images were set to the same scale before analysis using the Living Image Software (Perkin Elmer).

Quantification of fluorescent signal was carried out using the Living Image Software. Regions of interest (ROIs) were created automatically by the software and adjusted manually to cover each tissue piece individually (**Supplementary Figure 1A**). Signal is reported as total radiant efficiency, which is a unit less measure that uses photons per second then normalizes based on wattage and intensity of the excitation light. The average radiant efficiency value normalizes the total value by the size of each ROI.

### Fluorescence Microscopy of Tissues

OCT embedded tissue blocks were cryosectioned between 10-15 µM onto slides. Tissues were fixed for 15 minutes using 4% PFA in PBS, incubated with 0.5% Triton X-100 for 15 minutes to reduce background and non-specific antibody binding, then blocked in 5% BSA in PBS for 1 hour. Tissues were stained for SARS-CoV-2 using SARS-CoV-2 guinea pig antiserum (#NR-10361, BEI, USA) diluted in PBS 1:1000 overnight at 4°C. A goat anti-guinea pig secondary antibody labeled with AF647 or AF488 was diluted 1:500 in PBS and incubated for 1 hour at room temperature. All slides were Hoechst stained for 15 minutes (1:25,000, ThermoFisher, USA) and washed 3x with PBS between every step. Coverslips were mounted with Dako Fluorescence Mounting Medium (#S302380-2, Agilent, USA) and sealed with clear nail polish. Images were obtained using a DeltaVision Ultra inverted microscope (Cytivia, USA) using 10x, 20x, and 60x lenses. Images were deconvolved, stitched, and projected using softWoRx software (Applied Precision, USA).

### Image Analysis

Images were analyzed using the open-source image analysis software QuPath v0.3.0 (46). Tissue regions were annotated using the *Simple tissue detection* tool. To correlate the probe signal with the presence of infected cells, we segmented the nuclei using StarDist plugin (47) in QuPath v0.3.0, a state-of-the-art method outperforming classical approaches to detect star-convex objects for 2D images using a neural network. A total of 34 features were calculated for each cell in QuPath and mean intensity of respective image channel was used to identify the infected cells.

## Results

### Probe Selection and *In Vitro* Testing

**Figure 1:**
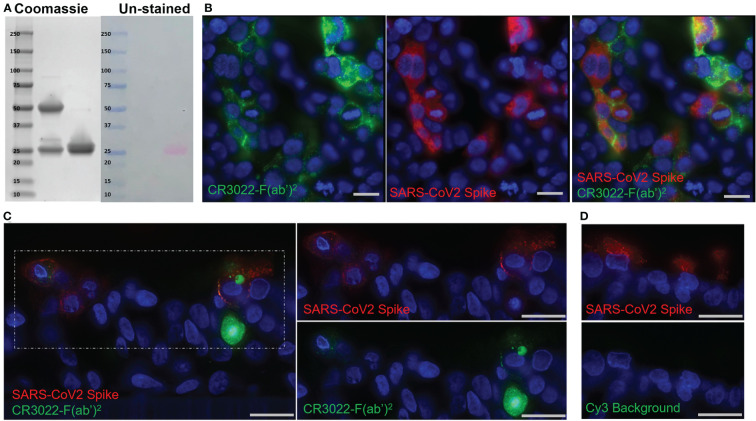
Development of the CR3022 F(ab’)2 Probe. **(A)** SDS-PAGE showing intact IgG (left) and F(ab’)2 (right) after digestion. Two bands around 25 kDa are seen in the F(ab’)2 lane indicating the light chain and digested heavy chains. Right side of image shows the same gel before staining with Simply-safe blue stain showing the labeling of the F(ab’)2 probe with the fluorophore Cy3. **(B)** Fluorescent microscopy images of 293T cells transfected with a SARS-CoV-2 spike protein plasmid. CR3022-F(ab’)2 labeled with Cy5 is shown in green and an anti-SARS-CoV-2 spike antibody is shown in red. **(C)** Fluorescent microscopy of SARS-CoV-2 infected human airway epithelium cultures. Cy3 labeled CR3022-F(ab’)2 shown in green, an anti-SARS-CoV-2 spike antibody shown in red, and the nuclear stain Hoechst shown in blue. **(D)** Images of an infected human airway epithelium culture showing lack of Cy3 background (green) in infected cells (red). Scale bars for all images 20µM.

We selected the monoclonal antibody (mAb) CR3022 that has been shown to bind to, but not efficiently neutralize, SARS-CoV-2 as the basis for our probe (48). CR3022 is an IgG1 isolated from an individual previously infected with SARS-CoV that binds a highly conserved portion of the receptor-binding domain of both the SARS-CoV and SARS-CoV-2 spike protein (48). To prepare the mAb for use as an *in vivo* probe, the Fc portion was removed through pepsin digestion to eliminate any Fc mediated localization of the intact IgG in live animals (**Figure 1A**). The resulting F(ab’)2 product was further modified by labeling with a fluorophore through an NHS-ester reaction (**Figure 1A**). We have previously shown that this labeling reaction has no effect on the ability of the IgG to engage its cognate antigen (43).

To validate the probe’s ability to bind to the SARS-CoV-2 spike on the surface of cells we performed an *in vitro* test using 293T cells that were transfected with a SARS-CoV-2 spike. Imaging confirmed the specificity of the Cy5 labeled probe to bind only cells that were expressing the SARS-CoV-2 spike (**Figure 1B**). Although we have used this methodology in the past with SIV infection, one concern was that unlike SIV, SARS-CoV-2 does not bud directly from the cell surface, likely leading to less spike protein being present on the cell membrane of infected cells *in vivo* (49, 50). However, there is evidence that infection with SARS-CoV-2 and other coronaviruses leads to a measurable amount of spike on the surface of infected cells (50–52). To confirm this and further validate the probe in a more physiologically relevant context, we tested the probe in primary human airway epithelium (HAE) cultures. These cultures consist of bronchial epithelial cells that are grown at an air liquid interface to induce differentiation into a ciliated, pseudostratified epithelium with tight junctions closely resembling the native conducting airway. Forty-eight hours p.i. the probe was applied to the basolateral surface of the HAE culture to mimic distribution of immunoglobulins *in vivo*. The probe was seen both on the surface of infected cells as well as intracellularly (**Figure 1C**). These data indicate that the probe can be internalized and accumulate in infected cells; thereby, increasing the power of the probe to detect infected cells. Infected cultures without probe showed no background fluorescence in the Cy3 channel (**Figure 1D**). Importantly, the finding that the probe was able to localize to the apical surface of the HAE after basolateral application implies that the probe can cross epithelial barriers like those of the lung, making it suited to detect infection in this compartment. These *in vitro* experiments showed that the probe specifically binds spike being expressed on the surface of cells and confirmed that there is enough spike on the plasma membrane of these differentiated cells for the probe to identify infected cells, thereby providing evidence for the use of the CR3022 based antibody probe in live animals infected with SARS-CoV-2.

### Characterization of the Fluorescently Labeled Probe in Infected Rhesus Macaques

**Figure 2:**
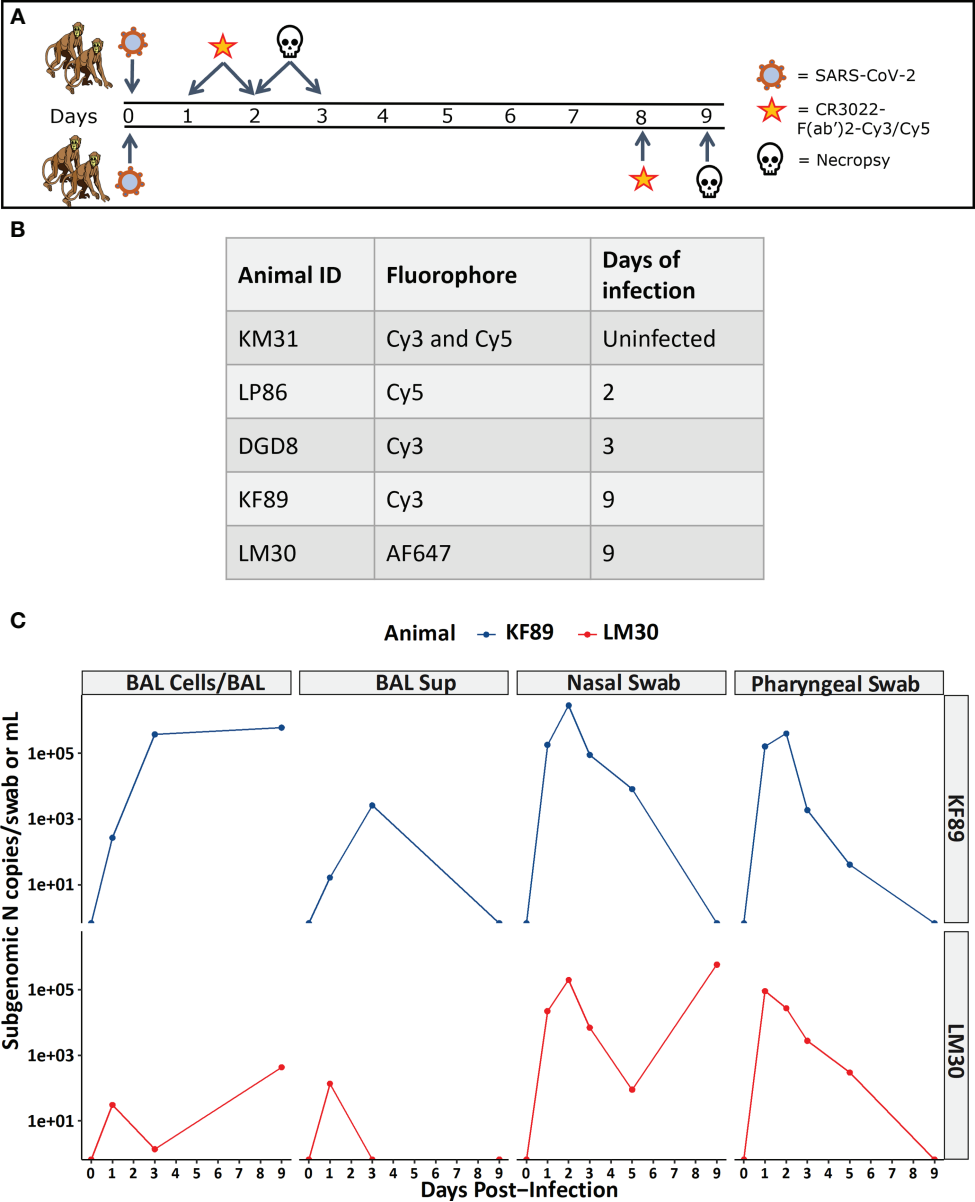
Study design and viral load measurements for infected macaques. **(A)** Infection and probe administration scheme for SARS-CoV-2 infected animals. **(B)** Table listing animal IDs and indicating the fluorophore that each individual animal received along with the day post-infection for necropsy. **(C)** Viral load data for 9-day infected animals measured as sub-genomic SARS-CoV-2 N mRNA from BAL cells, BAL supernatant, nasal swab, and pharyngeal swab.

**Figure 3:**
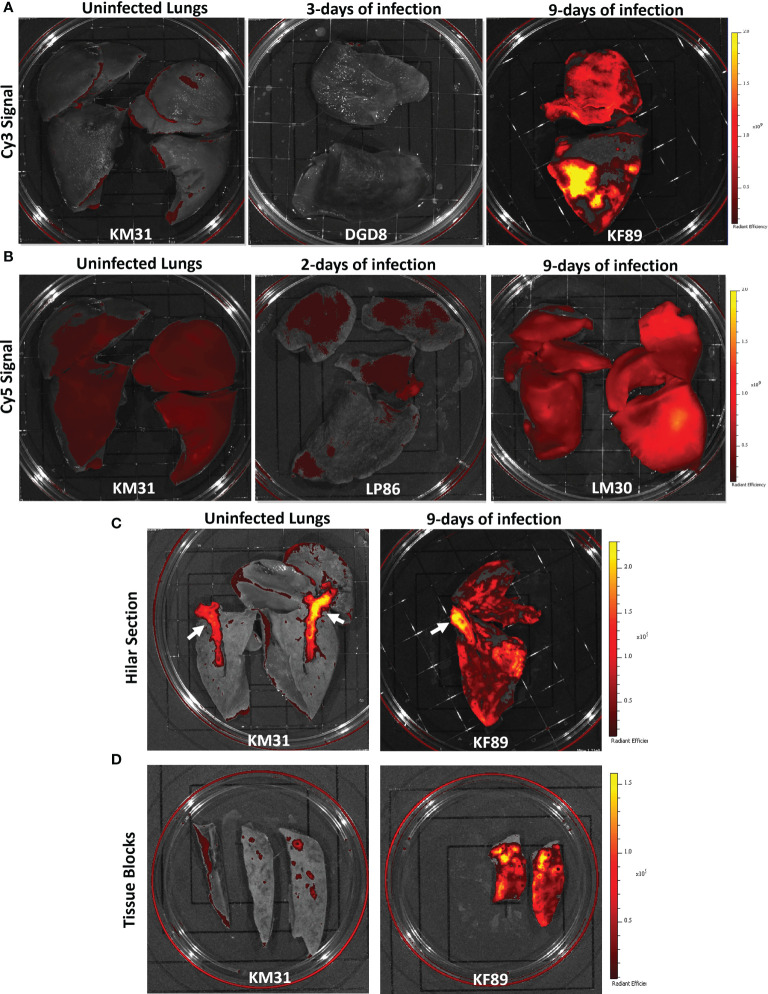
Fluorescent probe results from four SARS-CoV-2 infected rhesus macaques. **(A)** Whole lung IVIS images showing Cy3 fluorescent signal from three animals; KM31 (un-infected control), DGD8 (3-days of infection), and KF89 (9-days of infection). **(B)** Whole lung IVIS images showing Cy5 fluorescent signal from three animals; KM31 (un-infected control), LP86 (2-days of infection), and LM30 (9-days of infection). Scale is the same for each image. **(C)** Hilar section of left lower lung lobe from KM31 and KF89. **(D)** Approximately 2 cm by 4cm tissue blocks from the left lower lung lobe of KM31 and KF89.

To test the fluorescently labeled probes’ ability to disseminate and identify SARS-CoV-2 infection *in vivo* we utilized the rhesus macaque infection model. The probe was injected intravenously, and animals were necropsied 24 hours after probe administration as was shown to be optimal by previous studies (33, 42). Ongoing work to establish SARS-CoV-2 infection kinetics in rhesus macaques had shown that viral loads in bronchial alveolar lavage (BAL) fluid and nasopharyngeal swabs peak 2-3 days p.i. (15, 18, 19). Therefore, we hypothesized that this time point would give us the best opportunity to test the probes’ ability to detect infection in the lungs. The first two animals, LP86 and DGD8, were administered probe i.v. 1-day (LP86, Cy5) or 2-days (DGD8, Cy3) p.i. and were necropsied 24 hours later (**Figures 2A, B**). LP86 and DGD8 were administered probe labeled with different fluorophores, Cy5 and Cy3 respectively, to determine the optimal fluorophore in this system. To determine the background tissue and probe fluorescence associated with the lungs of an uninfected rhesus macaque we used a single uninfected animal (KM31) as a control for both fluorophores. This control animal was administered a mixture of the Cy3 and Cy5 probes 24 hours prior to necropsy (**Figure 2B**). Following necropsy and tissue fixation, lungs from all animals were fluorescently imaged at the whole organ level with IVIS imaging, which revealed no discernable difference in fluorescent signal between the control animal and the infected animals, regardless of fluorophore (**Figures 3A, B**). However, we did observe a higher background fluorescence signal when imaging in the Cy5 channel. Subsequent research has shown that the mAb CR3022 may have some ability to neutralize SARS-CoV-2 (53); therefore, we hypothesized that administering the probe early after infection may have changed the kinetics and spatial distribution of the infection resulting in a low number of infected cells. Consequently, to further test the probe and reduce the chance that it is interfering with infection we moved to a weeklong infection model.

For these studies, two additional animals (LM30, AF647 and KF89, Cy3) were administered probe i.v. 8 days after infection followed by necropsy 24 hours later (**Figures 2A** **–C**). Lungs were again fluorescently imaged using the same IVIS imaging settings as the previous animals. For LM30, we substituted Alexa Fluor 647, which can be imaged in the same channel as Cy5 to determine if the increased stability of the Alexa Fluor dyes compared to cyanine-based dyes could improve the signal of the probe. We found that after 9-days of infection, there was a strong Cy3 signal detected in the lungs of KF89 as compared to the lungs of KM31 and DGD8 (**Figure 3A**). Additionally, the detected signal was spread throughout the upper and lower lobes but appeared stronger in discrete areas and was not uniform across the entire tissue. These data corroborate pathology reports from other infection studies indicating that SARS-CoV-2 has a patchy, uneven distribution in the lungs (15, 16, 18–21). The AF647 signal in the lungs of LM30 was also higher than the Cy5 signal seen in the negative control animal, KM31 and the 2-day animal, LP86; however, the signal was more uniform across the lung tissue (**Figure 3A**). The higher Cy5 signal seen in uninfected control KM31, coupled with the unexpectedly uniform probe signal using two distinct fluorophores (Cy5 and AF647), led us to conclude that the background fluorescent signal in Cy5 was too high for straightforward interpretation of whole organ fluorescent images. In contrast, the Cy3 signal was seen in discrete areas in the lungs of KF89 after 9-days of infection, with little to no background signal, indicating that Cy3 is the optimal fluorophore for this system.

Furthermore, the differences in the Cy3 fluorescent signal between KM31 and KF89 persisted as the lungs were dissected. For example, a cut was made parallel to the frontal plane to bisect the hilum of the left lung and left main bronchi (**Figure 3C**). Fluorescent IVIS imaging of this dissection showed that the Cy3 signal persisted throughout the lung tissue of KF89 and was still absent from the bulk of the lung tissue from KM31. However, we did observe a high Cy3 fluorescent background associated with the hyaline cartilage of the main bronchi in both KM31 and KF89 (**Figure 3C**, white arrows). Further dissection of these tissues also illustrated that the signal in KF89 was present in tissue pieces at the centimeter scale and was clearly brighter and more prevalent than the signal in KM31 despite some background associated with the small airways (**Figure 3D**). These results indicate that the fluorescent probe accumulates in the lungs of animals that have more well-established infection.

### Comparison of Infected Cells Between Animals

**Figure 4:**
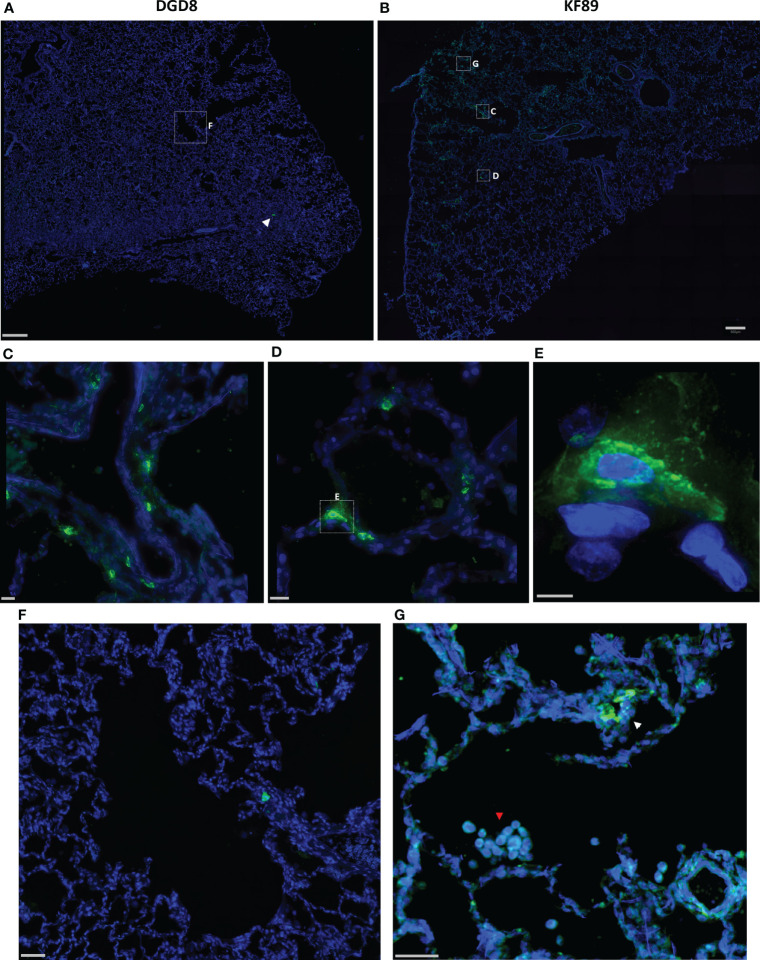
Fluorescent microscopy of infected cells in the lungs of DGD8 and KF89. **(A, B)** Representative low magnification (20x) fluorescent microscopy images of a section of lung from DGD8 **(A)** and KF89 **(B)**. SARS-CoV-2 anti-sera staining shown in green, Hoechst nuclear stain in blue. White arrowhead indicates small cluster of infected cells in DGD8. Scale bars 500µM. **(C, D)** Insets of fluorescent microscopy images of infected cells from the lungs of KF89. Scale bars 20µM. **(E)** High magnification image of infected Type 2 pneumocyte from lungs of KF89. Scale bar 5µM. **(F)** Insets of image of DGD8 lung showing two to three infected cells and no infiltration of immune cells. Scale bar 50µM **(G)** Inset of image of KF89 lung showing infected cells (white arrowhead) and infiltration of alveolar macrophages (red arrowhead). Scale bar 50µM.

**Figure 5:**
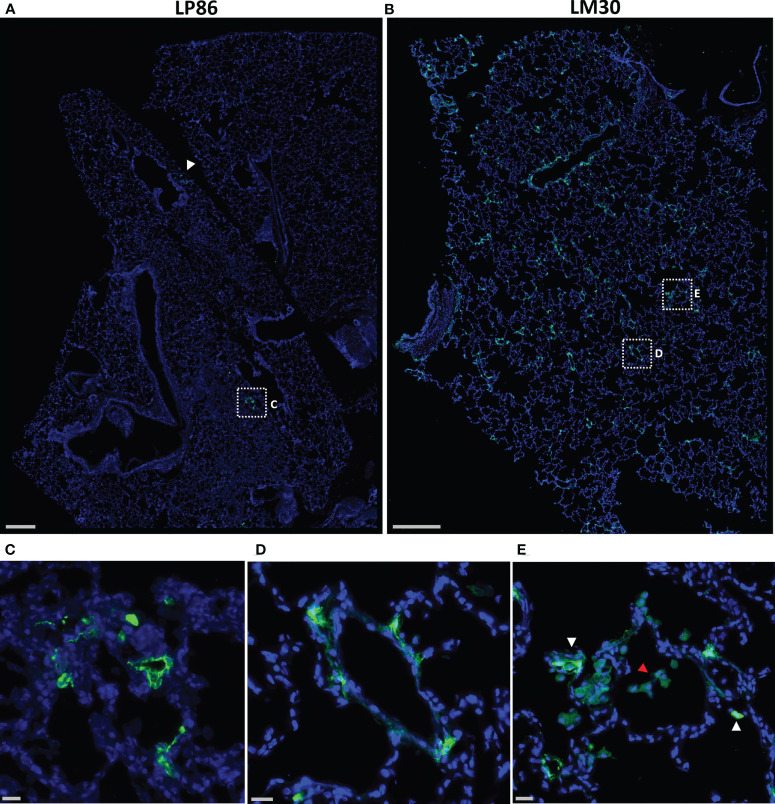
Fluorescent microscopy of infected cells in the lungs of LP86 and LM30. **(A, B)** Representative low magnification (20x) fluorescent microscopy images of a section of lung from LP86 **(A)** and LM30 **(B)**. SARS-CoV-2 anti-sera staining shown in green, Hoechst nuclear stain in blue. White arrowhead and box indicate two small clusters of infected cells in LP86. Scale bars 500µM. **(C, D)** Insets of fluorescent microscopy images of infected cells from LP86 **(C)** and LM30 **(D)**. Scale bars 20µM. **(E)** Inset of image of LM30 lung showing infiltration of alveolar macrophages (red arrowhead) and infected cells (white arrowheads) in alveolar septum. Scale bar 20µM.

To confirm the probes’ ability to locate infected cells in tissue, wide field fluorescent microscopy was used by staining with a SARS-CoV-2 antiserum. Large, low magnification (10x or 20x) image panels were acquired from multiple lung tissue sections for each animal to search for infected cells. Lung sections from KF89 showed SARS-CoV-2 infected cells distributed throughout the tissue (**Figure 4B**). In contrast, in DGD8 (**Figure 4A**) we observed only a few lung sections that contained small clusters of infected cells. These images validate the difference in infection between DGD8 and KF89 suggested by the Cy3 signal from the fluorescent probe. Furthermore, these results are consistent with our hypothesis that giving the probe only 1- or 2-days p.i. may have blocked some replication and led to a more limited infection. In addition, higher magnification images of KF89 show infected cells clustering near major airways (**Figure 4C**) as well as infected cells lining the alveolus (**Figure 4D**), including an infected type II pneumocyte (**Figure 4E**), recapitulating infection phenotypes previously reported in the rhesus macaque model (15–17, 20, 21, 23). Similar differences in infection were observed between LP86 and LM30 (**Figure 5**). LP86 had few infected cells, and several sections contained no infection (**Figures 5A, C**). In contrast, LM30 had robust infection with large numbers of infected cells widely distributed throughout lung sections (**Figure 5B**). Although the Cy5 and AF647 fluorescent signal had higher backgrounds, there was still a clear difference in fluorescent signal between LM30 and LP86 that was reflected by the difference in numbers of infected cells. Taken together, these data show that the fluorescently labeled probe can locate infected cells in lung tissue of rhesus macaques.

During our image analyses, we found that both 9-day animals, KF89 and LM30, also had substantial infiltration of immune cells, most likely alveolar macrophages, into the alveolar space of the lungs (**Figures 4G**, **5E**). These cells were not seen in the earlier time point animals, DGD8 and LP86 (**Figure 4F**). These alveolar macrophages have a small amount of signal in all channels, including the SARS-CoV-2 antiserum channel, the probe channel, and an unstained channel. While this is consistent with background autofluorescence, it is also possible that some of the antiserum and probe channel signals reflect real SARS-CoV-2 staining and probe distribution. It is possible that these alveolar macrophages may have picked up the probe through phagocytosis of infected cells or clearance of lysed but previously infected cells and subsequently increased the probe concentration at these sites (54).Although our images do not provide strong evidence for this, further work to understand whether the alveolar macrophages are contributing to accumulation of fluorescent probe signal is needed. Additionally, we see clear differences in the kinetics of infection between LM30 and KF89 despite both being necropsied 9 days p.i. LM30 has many more infected cells visible in the lungs while KF89 has fewer infected cells but substantially more alveolar macrophages present. Interestingly, neither animal had detectable viral load in the supernatant from a BAL collected at necropsy, but KF89 had a higher measured viral load in cells isolated from the same BAL (**Figure 2C**). This may reflect that KF89 was in the process of clearing the infection at the time of necropsy while LM30 still had active viral replication indicating slower infection kinetics, as seen by others (21). A detectable viral load in a nasal swab at necropsy for LM30, but not KF89, further corroborates this (**Figure 2C**). Because these two animals were given the probe labeled with different fluorophores, we cannot directly compare the fluorescent signals to see if this difference in kinetics in discernable by the probe. However, the results presented here show that the fluorescent probe signal we observed at the whole organ level mirrors the level of infection observed at the individual cell level. Next, we sought to determine if the probe can successfully discern between tissue pieces from a single animal containing differing levels of infected cells.

### Frequency of Infected Cells in Tissue From KF89

**Figure 6:**
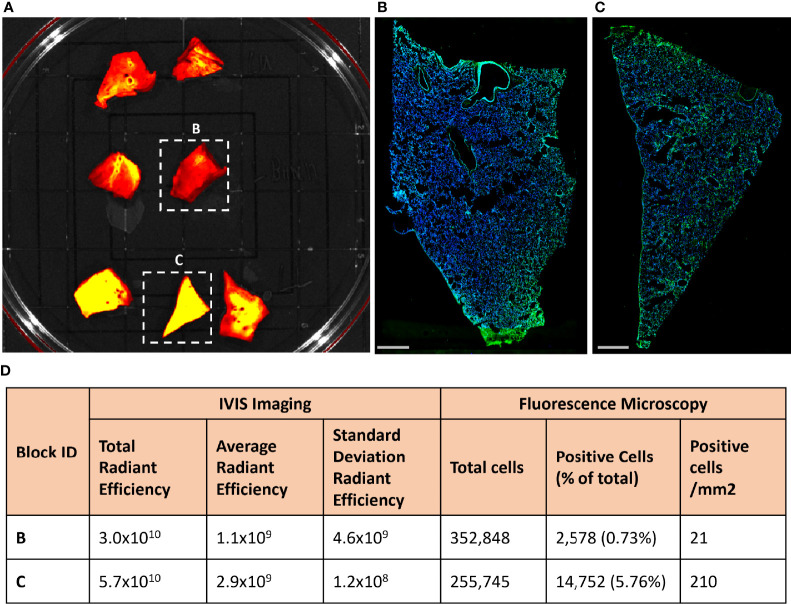
Fluorescent probe results from four SARS-CoV-2 infected rhesus macaques. **(A)** Approximately 2 cm by 2cm tissue blocks from the left lower lung of KF89 showing Cy3 fluorescent signal differences between tissues. **(B, C)** Microscopy images from indicated tissue blocks from **(A)**. Tissue was frozen in OCT, sectioned onto slides, and stained with SARS-CoV-2 anti-sera (green) and Hoechst nuclear stain (blue). Scale bars 2mm. **(D)** Values from quantification of IVIS fluorescence signal and infected cells from anti-sera staining.

To determine if the fluorescent signal in the lungs of KF89 could distinguish areas with high or low numbers of infected cells, 2cm by 2cm tissue blocks were cut from the left lower lung lobe of KF89 and fluorescently imaged by IVIS (**Figure 6A**). These tissue blocks displayed varying levels of Cy3 fluorescent signal, which was quantified for each block. The total radiant efficiency value and the average radiant efficiency value for two tissue blocks, one with high and one with low signal, is shown in **Figure 6D**. After IVIS imaging, these two blocks were frozen in OCT and cryo-sectioned onto slides. The tissue block that had the highest IVIS fluorescent signal correspondingly had the highest number of infected cells (**Figures 6C, D**) while the tissue block with less fluorescent signal had limited staining (**Figures 6B, D**). To quantify the staining, the total number of cells were counted for each tissue piece based on Hoechst staining of nuclei, followed by counting the number of these cells that had staining with the SARS-CoV-2 antiserum associated with them (**Supplementary Figure 1B, C**). These numbers show a notable difference in both the proportion and density of infected cells between these two tissue pieces (**Figure 6D**). These results indicate that the IVIS fluorescent probe signal can directly reflect the number of infected cells in tissue. Taken together, the results presented here show that the signal from the CR3022 probe can discern between animals with differing levels of infection and regions of high and low infection within an infected animal.

## Discussion

Antibody-based probes have been used extensively to study biological processes and have more recently gained traction in infectious disease research (25–28, 31–40, 42). The efficacy of these techniques for uncovering unknown sites of infection and elucidating the kinetics and distribution of viral infections has been proven in studies focusing on SIV pathogenesis in non-human primates (40, 42). The first such study, by Santangelo et al. (42), used PET/CT imaging combined with a radio-labeled antibody targeting the SIV envelope to show the reservoir sites of infected cells after initiation of anti-retroviral treatment in rhesus macaques. We and others have been using this same antibody probe to further study SIV pathogenesis and increase our understanding of the anatomical location of latently infected cells. After the emergence and spread of SARS-CoV-2 around the globe, we hypothesized that we could design an antibody-based probe to help further our knowledge of the pathogenesis and spatiotemporal distribution of this novel virus. We evaluated the potential of a fluorescently labeled F(ab’)2 fragment of the monoclonal antibody CR3022 in the pilot studies described here, toward the goal of developing it for future use as a PET/CT probe.

Here, we report the successful use of the mAb CR3022 as an *in vivo* probe targeting SARS-CoV-2 infection. *In vitro* testing demonstrated that the probe could label cells transfected with the spike protein as well as HAE cultures infected with a wild-type SARS-CoV-2 virus (**Figures 1B, C**). We infused the probe into infected rhesus macaques to determine if it could discern infection *in vivo*. However, we found very limited evidence of infection in lung tissue sections of these animals 2- or 3-days p.i. (**Figures 4A**, **5A**). Consequently, there was little to no fluorescent probe signal associated with the lungs of these animals (**Figure 3**). This contrasts with previous work in rhesus macaques showing that viral load in nasal swabs and BAL fluid peaked at day 2 or 3 p.i. and evidence of interstitial pneumonia and infection in lungs at 2 days p.i. (15, 18–20, 23). One reason we chose CR3022 as the basis for our probe is that it had been shown to bind tightly to, but not neutralize, SARS-CoV-2 (48). Not only has subsequent research shown that CR3022 has potential to neutralize SARS-CoV-2 (48), but we observed some inhibition of infection after applying the probe to the apical surface of an HAE culture early after infection (data not shown). Therefore, we hypothesize that the probe was interfering with the establishment and spread of infection in those macaques given probe 1- or 2-days p.i. Similar interference with the biological process under study has been seen with other antibody probes (34). This underscores the need to carefully choose antibodies to be developed as probes, as those that interfere with the natural progression of infections will be less useful in studying pathogenesis. We plan to test other antibodies in this system, as there has been an abundance of SARS-CoV-2 antibodies cloned from infected individuals since this study was conducted.

To minimize probe interference, we moved to a weeklong infection model to allow infection to become well established before administration of the probe. These animals were necropsied after 9-days of infection and showed strong probe-associated fluorescent signal in the lungs by IVIS imaging (**Figure 3**). Of note, the Cy3 labeled probe had less background fluorescence associated with the lung tissue as compared to the Cy5 or AF647 labeled probes. The difference in Cy3 fluorescent signal between the uninfected animal, KM31, and the animal infected for 9-days, KF89, persisted even as the lungs were dissected (**Figure 3D**). In the lungs of KF89 the fluorescent signal was also able to differentiate between two tissue pieces, one that contained a high number of infected cells and one that contained fewer cells based on immunofluorescence staining as well as quantification of signals (**Figure 6**). Importantly, the infection phenotype and kinetics elucidated by the probe matched what has been reported previously in the lungs of rhesus macaques (15, 16, 18–21, 23). This emphasizes that the probe signal is associated with physiologically relevant infection in the lungs and that giving the probe after infection was already established didn’t interfere with pathogenesis.

Microscopic analysis confirmed the difference in probe signal between animals infused acutely and those infused after 1 week of infection (**Figures 4** and **5**). The infected cells found in KF89 and LM30 were most often found in large foci. These large foci may be important for concentrating fluorescent probe signal and detection at the whole-organ level. We also observed clear differences in the kinetics of respiratory infection between KF89 and LM30 despite them receiving the same dose of virus and being necropsied at the same time. This manifested as a difference in both the number of infected cells and the frequency of alveolar macrophages. The presence of these alveolar macrophages in the lungs corroborates lung pathology previously established in the rhesus macaque model (20, 21). Although direct infection of alveolar macrophages was not observed, a weak signal in the probe channel indicates these cells may be acquiring the fluorescent probe through phagocytosis of excess probe or of previously infected dead cells labeled with the probe. Further work is necessary to determine what role macrophages are playing in the probe signal. Overall, the results observed here closely recapitulate the well-established respiratory tract infection in the rhesus macaque model (15–24). Taken together these data indicate that the CR3022 based *in vivo* probe works to elucidate sites of infected cells in the respiratory tract of SARS-CoV-2 infected rhesus macaques.

Although we chose to focus the early tests of this system on the respiratory tract, which is the most studied anatomical site of infection in macaques, to validate this technique for studying SARS-CoV-2, we are currently extending the use of this probe to studying whole animals using PET imaging. However, the use of PET scanners in biosafety level 3 animal facilities is limited and we wanted to prove this approach was successful using fluorescent imaging and a well-established anatomical site of infection before embarking on these resource intensive experiments. Previous work in mice has shown that signal from fluorescently labeled probes correlates well to signal from identical probes imaged using bioluminescence imaging (BLI) or PET (31, 34). The promising results reported here have given confidence in adopting this antibody-based fluorescent probe system to PET imaging. Performing PET imaging experiments using this probe will allow us to look at live animals to search for novel sites of infection in an unbiased manner possibly revealing new anatomical sites where SARS-CoV-2 expands and persists. Moreover, such a probe will allow us to study the kinetics of viral distribution in real time in a live animal. Notably, because these techniques use well established animal models and wild-type virus, this technique could be used to study and compare the pathogenesis of different SARS-CoV-2 variants and leveraged to test therapeutics in the rhesus macaque model as new studies have shown that CR3022 retains the ability to bind circulating variants (55).

This is the first report of an antibody based *in vivo* probe being used to study SARS-CoV-2 pathogenesis in non-human primates. Although a previous study examined the *in vivo* spread of a modified SARS-CoV-2 in mice expressing human ACE2 using BLI (56), using the antibody-based probe system has distinct advantages over BLI, which, relies on both a genetically modified pathogen and animal. In contrast, our antibody probe can be used in an unbiased manner and can be a powerful tool for uncovering novel sites of viral infection within a model that closely recapitulates human disease. In summary, we have demonstrated the utility of the CR3022 antibody-based probe system to locate sites of SARS-CoV-2 infection and to differentiate them from sites of no infection both at the whole organ and microscopic level.

## Data Availability Statement

The original contributions presented in the study are included in the article/**Supplementary Material**. Further inquiries can be directed to the corresponding author.

## Ethics Statement

The animal study was reviewed and approved by Tulane University Institutional Animal Care and Use Committee Bioqual, Inc. Institutional Animal Care and Use Committee.

## Author Contributions

PM and TH conceptualized the study and wrote the manuscript. PM, MA, MB, AC, MM, SX, CM, and LM-S performed experiments. RB, EP, and AD performed animal experiments. CR, MR, ML, J-PT, PA, and RV contributed animals and reagents and assisted with study design. SC contributed reagents. EM and FV contributed reagents and assisted with study design and administration. RLR assisted with data analysis. AC, MA, MB, FV, and RV critically reviewed the manuscript. All authors approved the manuscript before submission.

## Funding

This work was supported by National Institutes of Health grant R37 AI 094595-09S1 and in part by HHSN272201700033I (CR). This work was also supported in part by NIH grant OD011104. AC was supported in part by K01 OD026571-01.

## Conflict of Interest

Authors AD and ML are employed by Bioqual, Inc.

The remaining authors declare that the research was conducted in the absence of any commercial or financial relationships that could be construed as a potential conflict of interest.

## Publisher’s Note

All claims expressed in this article are solely those of the authors and do not necessarily represent those of their affiliated organizations, or those of the publisher, the editors and the reviewers. Any product that may be evaluated in this article, or claim that may be made by its manufacturer, is not guaranteed or endorsed by the publisher.
