# Supplementary material for: Development of an In Vivo Probe to Track SARS-CoV-2 Infection in Rhesus Macaques
Source: Front Immunol. 2021 Dec 24;12:810047. doi: 10.3389/fimmu.2021.810047 (PMC8739270; doi:10.3389/fimmu.2021.810047)
Supplement: Supplementary file 1 [file DataSheet_1.docx]

***Supplementary Material***


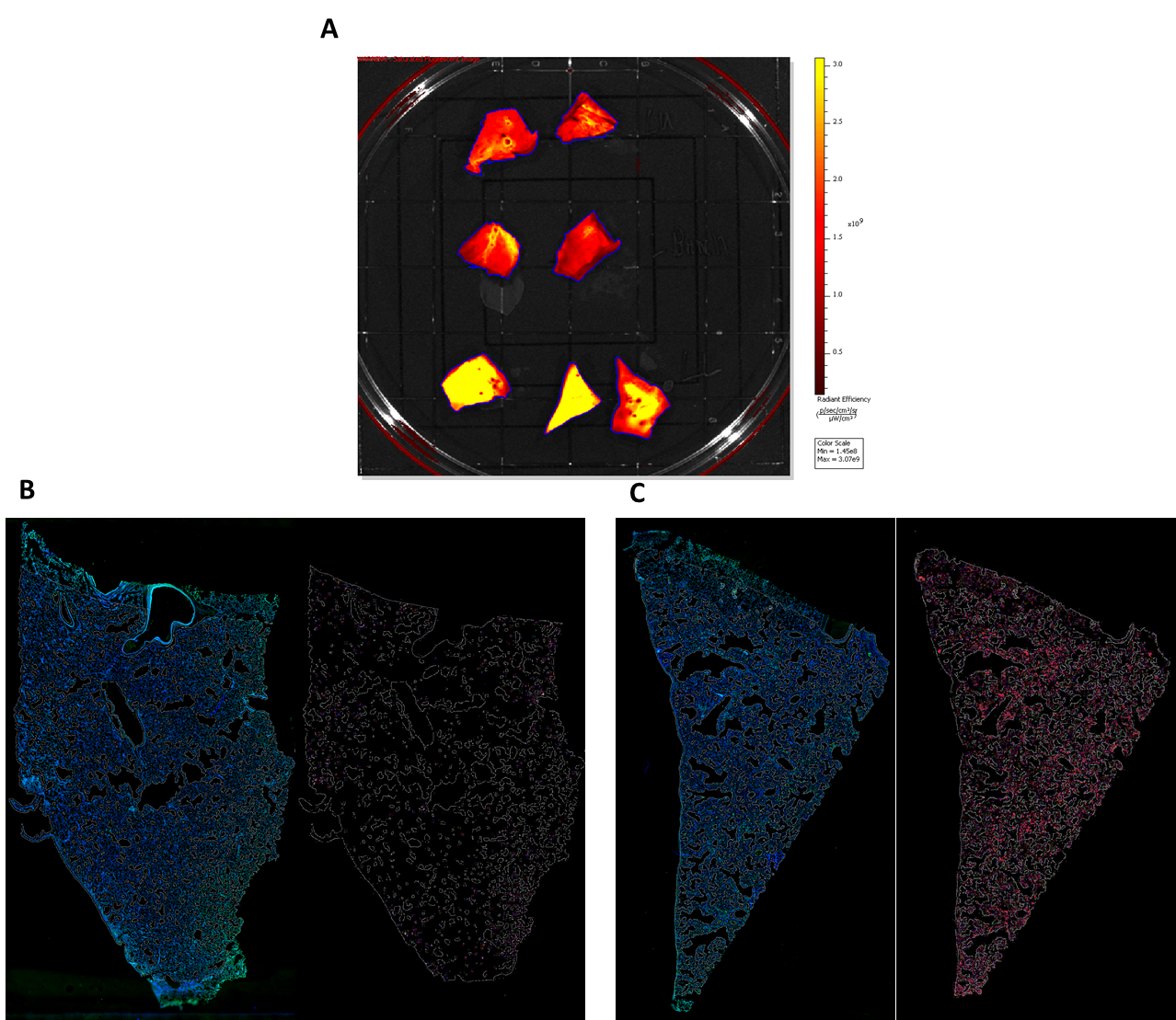


**Supplementary Figure 1.** Quantification of fluorescence signal and infected cells. **(A)** Fluorescence image of tissue blocks from KF89 showing ROIs (blue lines) used to quantify fluorescent signal. **(B and C)** Images used for quantifying infected cells. Each blue dot represents a single cell and those that are red indicate infected cells. White lines show the area included in the analysis, the top and bottom edges of each tissue block were excluded from analysis due to autofluorescence.
